# Dynamic metabolic responses of brown planthoppers towards susceptible and resistant rice plants

**DOI:** 10.1111/pbi.12721

**Published:** 2017-04-13

**Authors:** Caixiang Liu, Ba Du, Fuhua Hao, Hehua Lei, Qianfen Wan, Guangcun He, Yulan Wang, Huiru Tang

**Affiliations:** ^1^ CAS Key Laboratory of Magnetic Resonance in Biological Systems State Key Laboratory of Magnetic Resonance and Atomic and Molecular Physics National Centre for Magnetic Resonance in Wuhan Wuhan Institute of Physics and Mathematics, the Chinese Academy of Sciences Wuhan China; ^2^ State Key Laboratory of Hybrid Rice College of Life Sciences Wuhan University Wuhan China; ^3^ State Key Laboratory of Genetic Engineering Zhongshan Hospital and School of Life Sciences Fudan University Collaborative Innovation Center for Genetics and Development Metabonomics and Systems Biology Laboratory at Shanghai International Centre for Molecular Phenomics Shanghai China; ^4^ Collaborative Innovation Center for Diagnosis and Treatment of Infectious Diseases Zhejiang University Hangzhou China

**Keywords:** brown planthopper, resistant and susceptible rice, metabonomics, real‐time PCR, GC‐FID/MS

## Abstract

Brown planthopper (*Nilaparvata lugens* Stål, BPH) causes huge economic losses in rice‐growing regions, and new strategies for combating BPH are required. To understand how BPHs respond towards BPH‐resistant plants, we systematically analysed the metabolic differences between BPHs feeding on the resistant and susceptible plants using NMR and GC‐FID/MS. We also measured the expression of some related genes involving glycolysis and biosyntheses of trehalose, amino acids, chitin and fatty acids using real‐time PCR. BPH metabonome was dominated by more than 60 metabolites including fatty acids, amino acids, carbohydrates, nucleosides/nucleotides and TCA cycle intermediates. After initial 12 h, BPHs feeding on the resistant plants had lower levels of amino acids, glucose, fatty acids and TCA cycle intermediates than on the susceptible ones. The levels of these metabolites recovered after 24 h feeding. This accompanied with increased level in trehalose, choline metabolites and nucleosides/nucleotides compared with BPH feeding on the susceptible plants. Decreased levels of BPH metabolites at the early feeding probably resulted from less BPH uptakes of sap from resistant plants and recovery of BPH metabolites at the later stage probably resulted from their adaptation to the adverse environment with their increased hopping frequency to ingest more sap together with contributions from yeast‐like symbionts in BPHs. Throughout 96 h, BPH feeding on the resistant plants showed significant up‐regulation of chitin synthase catalysing biosynthesis of chitin for insect exoskeleton, peritrophic membrane lining gut and tracheae. These findings provided useful metabolic information for understanding the BPH–rice interactions and perhaps for developing new BPH‐combating strategies.

## Introduction

Rice (*Oryza sativa* L.) is a primary food crop and often attacked by various insect pests. Among those, the brown planthopper (*Nilaparvata lugens* Stål, BPH) is one of the most serious pests, which ingests sap from the phloem of rice using its stylets, causing huge yield losses. BPH damage causes over one billion kilogram losses in rice yield every year in China alone (Cheng *et al*., 2013). In rice‐growing regions, the most popular way to control BPH is the use of chemical insecticides. However, insecticide abuse can cause serious environmental problems and BPH resistance towards insecticides (Lakshmi *et al*., 2010; Nagata, 1984). An alternative way for combating the BPH problem is to develop BPH‐resistant rice varieties. Some rice germplasms exhibit resistance to BPH as they carry BPH‐resistant genes (Pathak *et al*., 1969). When attacked by BPH, resistant rice plants suffered little damage and grow normally, whereas the survival rate of BPH nymph is significantly compromised (Alagar *et al*., 2007; Alam and Cohen, 1998; Wang *et al*., 2008). Therefore, it is widely accepted that breeding and growing BPH‐resistant rice plants is the most effective and environment‐friendly method to control BPH pest. Understanding the interactions between rice plants and BPH will offer potentially vital information for developing new BPH‐resistant rice varieties and/or BPH‐control strategies.

The BPH–rice interactions are complex, and previous studies focused on mainly the responses of rice to BPH attacks. The study by Hao *et al*. (2008) indicated that BPH feeding induced callose to deposit on sieve plates of resistant rice phloem, preventing insects from ingesting sap. BPH infestation also induced higher levels of trypsin inhibitor together with callose deposition in *Bph14*‐expressing resistant lines (Du *et al*., 2009). Research results further showed that plants expression *Bph26,* another BPH‐resistant gene, induced callose deposition in rice phloem sieve tube as well (Tamura *et al*., 2014). Proteomics results indicated that oxidative stress response proteins, β‐glucanases, kinases, photosynthesis proteins and aquaporins, had significant alterations in rice upon BPH infection (Wei *et al*., 2009). Metabonomics results suggested that the activation of both the shikimate‐mediated secondary metabolism and GABA shunt was crucial for rice to defend against BPH attack (Liu *et al*., 2010). Furthermore, different rice plants also cause different responses from BPH metabolism. For example, BPH feeding on moderate resistant rice variety, Mudgo carrying *bph1*, showed up‐regulations of genes involved in the metabolism of amino sugars and nucleotide sugars, as well as carbohydrate digestion and absorption, compared with BPH feeding on susceptible rice variety TN1 (Ji *et al*., 2013). When BPH transferred from the susceptible rice TN1 to the resistant rice B5 (expressing *Bph14* and *Bph15*), 27 genes involved in the syntheses of trehalose, ribonucleoside hydrolase and triacylglycerol lipase were up‐regulated (Wang *et al*., 2015). Proteomic changes have been noted in BPH feeding on different rice plants (Huang *et al*., 2016; Konishi *et al*., 2009; Sharma *et al*., 2004). For example, the saliva of BPH reared on the susceptible rice variety (Xiushui 134) contains more abundant proteins in digestion function (Huang *et al*., 2016). However, these investigations were only concentrated on the impact of different feedings on BPH metabolisms in terms of gene and protein expressions. The metabolic responses of BPH to susceptible and resistant rice feedings remain poorly understood. Recently, Peng *et al*. (2016) reported metabonomic differences between the biotype 1 BPH honeydew feeding on the susceptible rice TN1 and the resistant rice YHY15 for 48 h (Peng *et al*., 2016). They found that the BPH honeydew feeding on YHY15 had higher levels for two TCA cycle intermediates (succinate and malate) and urea but lower levels for six amino acids (Val, Leu, Ser, Thr, Pro and Gln) (Peng *et al*., 2016). This suggests that the metabolic effects of infecting the resistant and susceptible rice lines on the BPH metabonome may be different as well although such data are not yet available.

It is worth noting that BPH harbours yeast‐like symbionts (YLS) mainly in mycetocytes (Chen *et al*., 1981; Cheng *et al*., 2013; Pang *et al*., 2012). These YLS can supply essential nutrients for BPH under nutrient deprivation conditions (Chen *et al*., 2011b; Lu *et al*., 2004) that may play important roles in the BPH–rice interaction. For example, YLS can provide their insect host with some essential amino acids, sterols and vitamins (Baumann, 2005; Xue *et al*., 2014).

Metabonomics has become a powerful tool for understanding the dynamic metabolic responses of a given biological system to both endogenous and exogenous factors (Fiehn, 2002; Nicholson *et al*., 1999; Tang and Wang, 2006). Such an approach has been applied to studying the metabolic responses of rice to BPH feeding (Liu *et al*., 2010; Uawisetwathana *et al*., 2015), of chickpea to *Fusarium oxysporum* infection (Kumar *et al*., 2015) and of plants towards osmotic stresses (Dai *et al*., 2010; Foito *et al*., 2009; Zhang *et al*., 2011). However, the metabolic responses of BPH towards ingesting different rice remain largely vague although such information is conceivably essential for developing new BPH‐resistant rice varieties.

In this work, we systematically analysed the differential metabolic responses of BPH feeding on susceptible and resistant rice plants at various time points using the NMR‐based metabonomics approach. We also measured the dynamic fatty acid profiles of these BPHs using GC‐FID/MS. We further evaluated the expression levels of several related genes by real‐time PCR so as to consolidate our findings obtained from metabonomics. The results provided a comprehensive overview of BPH metabolic responses towards feeding on the BPH‐susceptible and BPH‐resistant rice plants.

## Results

### Metabolite compositions of BPH nymphs

**Figure 1:**
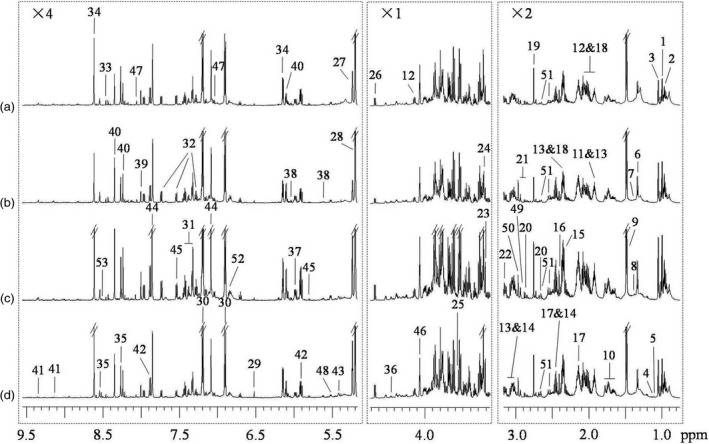
^1^H NMR spectra of BPH nymph extracts feeding on (a) resistant rice plants for 12 h, (b) susceptible plants for 12 h, (c) resistant rice plants for 96 h and (d) susceptible plants for 96 h. The regions δ 0.75–3.17 and δ 5.17–9.55 were vertically expanded two times and four times, respectively. Key: 1, isoleucine (Ile); 2, leucine (Leu); 3, valine (Val); 4, 2‐ketoisovalerate; 5, 3‐methylaspartate; 6, threonine (Thr); 7, 2‐hydroxyisobutyrate; 8, lysine (Lys); 9, alanine (Ala); 10, arginine (Arg); 11, acetate; 12, proline (Pro); 13, γ‐aminobutyrate (GABA); 14, α‐ketoglutarate (α‐KG); 15, pyruvate; 16, succinate; 17, glutamine (Gln); 18, glutamate (Glu); 19, dimethylamine; 20, aspartate (Asp); 21, asparagine (Asn); 22, ethanolamine (EA); 23, choline; 24, glycerophosphocholine (GPC); 25, glycine (Gly); 26, β‐glucose; 27, α‐glucose; 28, trehalose; 29, fumarate; 30, tyrosine (Tyr); 31, phenylalanine (Phe); 32, tryptophan (Trp); 33, formate; 34, adenosine monophosphate (AMP); 35, adenosine diphosphate/adenosine triphosphate (ADP/ATP); 36, uridine monophosphate (UMP); 37, cytidine monophosphate (CMP); 38, uridine diphosphate glucose (UDP‐glucose); 39, guanosine; 40, inosine; 41, nicotinamide adenine dinucleotide (NAD); 42, uridine; 43, allantoin; 44, histidine (His); 45, uracil; 46, myo‐inositol; 47, carnosine; 48, uridine diphosphate‐N‐acetyl glucosamine; 49, dimethylglycine; 50, unknown (U1); 51, citrate; 52, unknown (U2); 53, deoxyadenosine monophosphate (dAMP).

^1^H NMR spectra of BPH nymph extracts (Figure 1) showed obvious metabolite differences between BPH nymphs feeding on the resistant and susceptible rice plants for 12 h and 96 h. Both ^1^H and ^13^C signals were assigned to various metabolites (Table S1) using literature data (Dai *et al*., 2010; Fan, 1996; Fan and Lane, 2008) together with the in‐house developed and publically accessible databases (Cui *et al*., 2008). Identities of these metabolites were further confirmed by a series of 2D NMR spectra as always required in this laboratory. From the extracts of BPH nymphs, over 60 metabolites were unambiguously identified including amino acids and their metabolites, sugars, organic acids, metabolites of nucleosides and choline (Figure 1, Table S1) together with 16 fatty acids (Table S2).

Visual inspection of these spectra suggested that BPH feeding on resistant rice plants for 12 h had lower levels of inosine, Thr and Trp than those feeding on susceptible rice plants for the same period (Figure 1a–b). The BPH nymphs feeding on the resistant rice plants for 96 h had higher levels of citrate and His than those feeding on the susceptible rice plants for the same duration (Figure 1c–d). To substantiate these observations, we have quantified concentration of the abundant metabolites in BPH nymphs feeding on different rice plants (Table 1), which has not been reported previously to the best of our knowledge. Among these BPH metabolites, Gln and trehalose have the highest abundance (about 0.9–2.2 mg per gram fresh weight BPH) followed by Ala, Pro, Tyr, glucose, choline and AMP (about 0.3–1.4 mg/g). The contents of fumarate, formate and guanosine were all below 0.1 mg/g. These quantitative results confirmed the above observations on inosine, Thr and Trp.

**Table 1:** Quantitative results for metabolites in BPH nymphs

Metabolites	δ (Η)	T1 (S)	Metabolite quantity^a^ (Mean ± SD, mg/g fresh weight BPH nymphs)							
			R12h^b^	S12h^b^	R24h	S24h	R48h	S48h	R96h	S96h
Ile	1.01	0.93	0.125 ± 0.033	0.158 ± 0.022	0.170 ± 0.021	0.134 ± 0.018	0.151 ± 0.032	0.137 ± 0.040	0.206 ± 0.039	0.196 ± 0.060
Val	1.04	0.98	0.167 ± 0.037	0.193 ± 0.032	0.212 ± 0.030	0.181 ± 0.038	0.161 ± 0.054	0.158 ± 0.042	0.232 ± 0.053	0.238 ± 0.074
Thr	1.33	0.90	0.218 ± 0.047	0.231 ± 0.022	0.199 ± 0.038	0.207 ± 0.047	0.218 ± 0.039	0.169 ± 0.037	0.250 ± 0.051	0.263 ± 0.060
Ala	1.48	1.46	0.794 ± 0.196	0.747 ± 0.098	0.556 ± 0.128	0.516 ± 0.108	0.772 ± 0.152	0.753 ± 0.186	0.809 ± 0.152	0.884 ± 0.282
Pro	4.14	5.44	0.725 ± 0.215	0.646 ± 0.109	0.512 ± 0.113	0.512 ± 0.093	0.708 ± 0.171	0.486 ± 0.190	0.867 ± 0.184	0.907 ± 0.246
Gln	2.14	1.11	1.422 ± 0.406	1.185 ± 0.198	1.171 ± 0.275	1.142 ± 0.230	1.368 ± 0.314	0.897 ± 0.315	1.689 ± 0.275	1.542 ± 0.493
Asn	2.96	1.34	0.208 ± 0.073	0.227 ± 0.032	0.223 ± 0.049	0.233 ± 0.045	0.324 ± 0.073	0.213 ± 0.077	0.371 ± 0.057	0.371 ± 0.133
Tyr	6.90	2.83	0.419 ± 0.111	0.412 ± 0.106	0.418 ± 0.103	0.389 ± 0.107	0.345 ± 0.095	0.364 ± 0.076	0.420 ± 0.086	0.466 ± 0.155
Phe	7.43	2.85	0.080 ± 0.025	0.090 ± 0.013	0.089 ± 0.021	0.080 ± 0.018	0.097 ± 0.024	0.068 ± 0.027	0.120 ± 0.019	0.129 ± 0.046
Trp	7.73	1.00	0.080 ± 0.023	0.099 ± 0.016	0.095 ± 0.021	0.089 ± 0.018	0.105 ± 0.027	0.086 ± 0.030	0.127 ± 0.023	0.115 ± 0.046
Asp	2.82	1.34	0.240 ± 0.074	0.220 ± 0.035	0.186 ± 0.039	0.190 ± 0.035	0.283 ± 0.064	0.271 ± 0.063	0.325 ± 0.051	0.336 ± 0.108
Succinate	2.41	2.12	0.146 ± 0.023	0.112 ± 0.016	0.093 ± 0.022	0.085 ± 0.015	0.073 ± 0.021	0.122 ± 0.025	0.135 ± 0.026	0.160 ± 0.049
Formate	8.46	13.72	0.011 ± 0.003	0.011 ± 0.002	0.011 ± 0.003	0.012 ± 0.004	0.017 ± 0.002	0.008 ± 0.002	0.012 ± 0.003	0.014 ± 0.004
Citrate	2.55	0.68	0.263 ± 0.068	0.299 ± 0.044	0.325 ± 0.077	0.334 ± 0.071	0.392 ± 0.087	0.257 ± 0.097	0.471 ± 0.084	0.435 ± 0.161
Fumarate	6.52	11.41	0.009 ± 0.005	0.008 ± 0.003	0.011 ± 0.006	0.013 ± 0.006	0.010 ± 0.009	0.016 ± 0.012	0.023 ± 0.008	0.023 ± 0.013
β‐Glucose	4.65	1.62	0.479 ± 0.133	0.504 ± 0.065	0.337 ± 0.096	0.283 ± 0.065	0.326 ± 0.135	0.358 ± 0.155	0.666 ± 0.141	0.842 ± 0.270
α‐Glucose	5.24	3.31	0.352 ± 0.089	0.386 ± 0.050	0.257 ± 0.072	0.218 ± 0.048	0.249 ± 0.096	0.232 ± 0.116	0.491 ± 0.103	0.627 ± 0.193
Trehalose	5.20	1.25	1.415 ± 0.322	1.356 ± 0.297	1.783 ± 0.524	1.630 ± 0.432	1.933 ± 0.497	1.398 ± 0.539	2.215 ± 0.473	1.747 ± 0.827
AMP	8.61	2.15	0.550 ± 0.161	0.558 ± 0.063	0.406 ± 0.06	0.341 ± 0.055	0.489 ± 0.106	0.321 ± 0.108	0.628 ± 0.127	0.612 ± 0.168
UDP‐glucose	7.96	1.00	0.203 ± 0.065	0.187 ± 0.027	0.185 ± 0.046	0.198 ± 0.049	0.211 ± 0.054	0.149 ± 0.055	0.280 ± 0.054	0.239 ± 0.093
Inosine	8.35	2.41	0.208 ± 0.015	0.248 ± 0.025	0.248 ± 0.06	0.249 ± 0.053	0.298 ± 0.073	0.204 ± 0.083	0.370 ± 0.059	0.372 ± 0.161
Uridine	7.88	1.41	0.178 ± 0.056	0.160 ± 0.025	0.151 ± 0.025	0.128 ± 0.023	0.183 ± 0.043	0.120 ± 0.044	0.216 ± 0.031	0.237 ± 0.085
Guanosine	8.01	1.00	0.074 ± 0.024	0.062 ± 0.012	0.051 ± 0.012	0.056 ± 0.013	0.067 ± 0.017	0.072 ± 0.021	0.090 ± 0.018	0.091 ± 0.037
Ethanolamine	3.15	1.20	0.140 ± 0.044	0.169 ± 0.025	0.154 ± 0.034	0.120 ± 0.030	0.201 ± 0.043	0.136 ± 0.049	0.237 ± 0.040	0.236 ± 0.073
Choline	3.20	1.58	0.426 ± 0.154	0.420 ± 0.085	0.436 ± 0.078	0.330 ± 0.068	0.465 ± 0.111	0.332 ± 0.137	0.646 ± 0.155	0.637 ± 0.163

a In the form of mg/g fresh weight BPH nymphs, mean ± SD.

b R12h and S12h represented metabolites in BPH nymphs feeding on resistant and susceptible rice plants for 12 h, respectively. Data in red and blue, respectively, denote significant increase and decrease in BPH feeding on the resistant rice plants compared with those feeding on the susceptible rice plants (*P* < 0.05).

### Metabolic phenotype differences between BPH nymphs feeding on the susceptible and resistant rice plants

**Figure 2:**
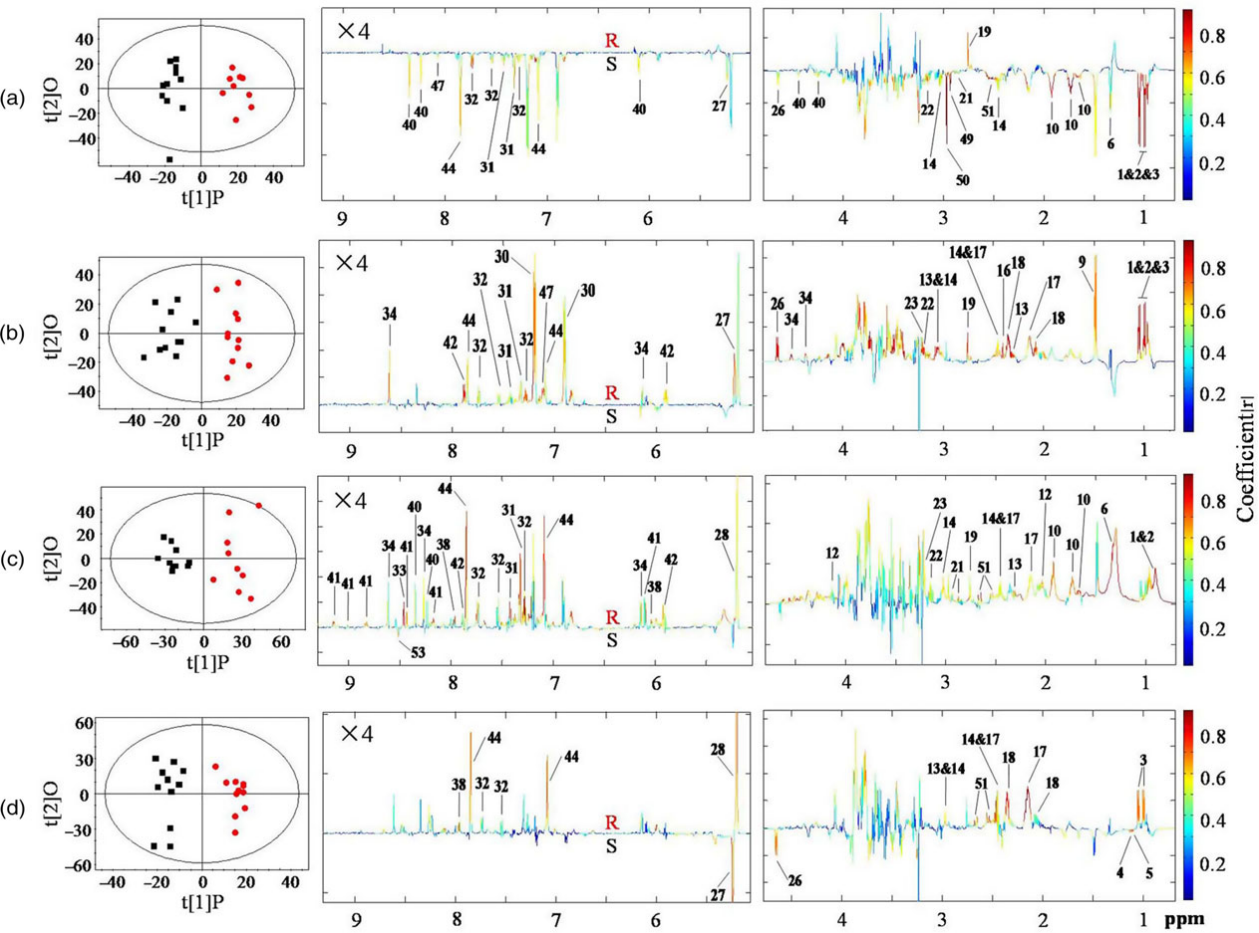
OPLS‐DA scores plots (left) and coefficient‐coded loadings plots (right) showing time dependence of *Bph15* effects on BPH metabolism between BPH nymphs feeding on resistant rice plants (red dot, 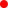) and ones feeding on susceptible lines (black box, ■) for (a) 12 h (R^2^X = 0.357, *Q* ^2^ = 0.826, *P *=* *0.00001), (b) 24 h (R^2^X = 0.379, *Q* ^2^ = 0.728, *P *=* *0.0001), (c) 48 h (R^2^X = 0.578, *Q* ^2^ = 0.738, *P *=* *0.0005) and (d) 96 h (R^2^X = 0.366, *Q* ^2^ = 0.757, *P *=* *0.00004). Metabolite keys are the same as in Figure 1 and Table S1.

Principal component analysis (PCA) of the NMR data showed clear metabolic differences between BPH nymphs feeding on the resistant and susceptible rice plants at four different time points (Figure S1). Orthogonal projection to latent structure‐discriminant analysis (OPLS‐DA) further verified such observation (Figure 2, Table 2). The coefficient‐coded loadings plots of OPLS‐DA showed that BPH nymphs feeding on the resistant rice plants for 12 h had significantly lower levels of glucose, organic acids, inosine, ethanolamine and most of amino acids than those feeding on the susceptible plants (Figure 2a, Table 2). After feeding for 24 h and 48 h, however, nearly all these down‐regulated metabolites had higher levels in BPH nymphs feeding on the resistant plants than in those feeding on susceptible plants (Figure 2b, Table 2). Furthermore, the levels of trehalose, formate, citrate, inosine, NAD and UDP‐glucose were also higher in BPH feeding on resistant plants for 48 h (Figure 2c, Table 2). BPH nymphs feeding on resistant plants for 96 h showed significantly higher levels of six amino acids (Val, GABA, Gln Glu, Trp and His), trehalose, α‐ketoglutarate, citrate and UDP‐glucose but lower levels of glucose, 3‐methylaspartate and 2‐ketoisovalerate than BPH feeding on susceptible plants (Figure 2d, Table 2). Such intergroup differences were conformable with quantitative data for BPH metabolites at four different time points (Table 1).

**Table 2:** The metabolite changes in BPH nymphs feeding on the resistant rice plants compared with ones feeding on the susceptible lines

Metabolites (keys)	Coefficient (*r*)^a^			
Amino acids	R12h vs S12h	R24h vs S24h	R48h vs S48h	R96h vs S96h
Ile (1)^b^	−0.882^c^	0.759	0.805	
Leu (2)	−0.872	0.764	0.770	
Val (3)	−0.917	0.828		0.714
3‐Methylaspartate (4)				−0.781
Thr (6)	−0.778		0.836	
Ala (9)		0.709		
Arg (10)	−0.924		0.779	
Pro (12)			0.799	
GABA (13)		0.867	0.805	0.597
Gln (17)		0.865	0.599	0.802
Glu (18)		0.855		0.768
Asn (21)	−0.783		0.823	
Tyr (30)		0.673		
Phe (31)	−0.661	0.677	0.793	
Trp (32)	−0.882	0.732	0.843	0.580
His (44)	−0.779	0.672	0.919	0.655
Carnosine (47)	−0.646	0.583		
Sugars				
Glucose (26,27)	−0.675	0.869		−0.686
Trehalose (28)			0.658	0.659
Organic acids				
2‐Ketoisovalerate (5)				−0.793
α‐Ketoglutarate (14)	−0.745	0.698	0.615	0.736
Succinate (16)		0.828		
Formate (33)			0.950	
Citrate (51)	−0.948		0.750	0.790
Nucleotide metabolites				
AMP (34)		0.782	0.700	
UDP‐glucose (38)			0.912	0.757
Inosine (40)	−0.709		0.571	
NAD (41)			0.784	
Uridine (42)		0.848	0.786	
dAMP (53)			−0.692	
Choline metabolites				
Dimethylamine (19)	0.777	0.788	0.660	
Ethanolamine (22)	−0.846	0.880	0.924	
Choline (23)		0.657	0.638	
Dimethylglycine (49)	−0.957			

a The coefficients were obtained from OPLS‐DA results, and positive and negative signs indicate positive and negative correlation in the concentrations, respectively.

b Metabolite keys are in Figure 1.

c Positive and negative signs indicate the increase and decrease in the metabolites. Values for *P* ≥ 0.05 were not tabulated. R12h and S12h denote metabolites in BPH nymphs feeding on the resistant and susceptible rice plants for 12 h, respectively.

**Figure 3:**
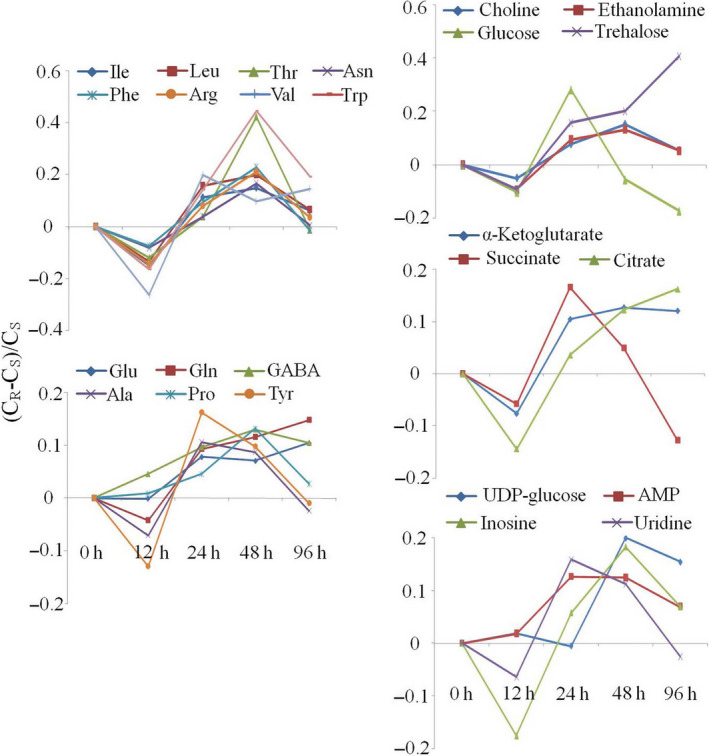
The ratios of concentration for metabolites in BPH nymphs feeding on resistant rice plants against those feeding on susceptible lines. Metabolites with statistically significant variations are listed in Figure 2 and Table 2.

The intergroup differences for BPH were also displayed in the forms of metabolite concentration ratios, (*C*_*R*_–*C*_*S*_)/*C*_*S*_, as a function of feeding time, where *C*_*R*_ and *C*_*S*_ stood for the metabolite concentrations in BPH nymphs feeding on resistant and susceptible plants, respectively (Figure 3). The results indicated that compared with those in BPH feeding on susceptible plants, the levels of most metabolites were decreased by 10%–20% at 12 h and gradually increased to reach maximum at 48 h or 96 h. Some metabolites remained at high levels even after 96 h of feeding, including GABA, glutamine, glutamate, trehalose, α‐ketoglutarate, citrate and UDP‐glucose. Among them, trehalose level was 40% higher in BPH nymphs feeding on the resistant rice plants than in those feeding on the susceptible rice plants (Figure 3).

### Fatty acid differences in BPH nymphs feeding on the susceptible and resistant plants

**Figure 4:**
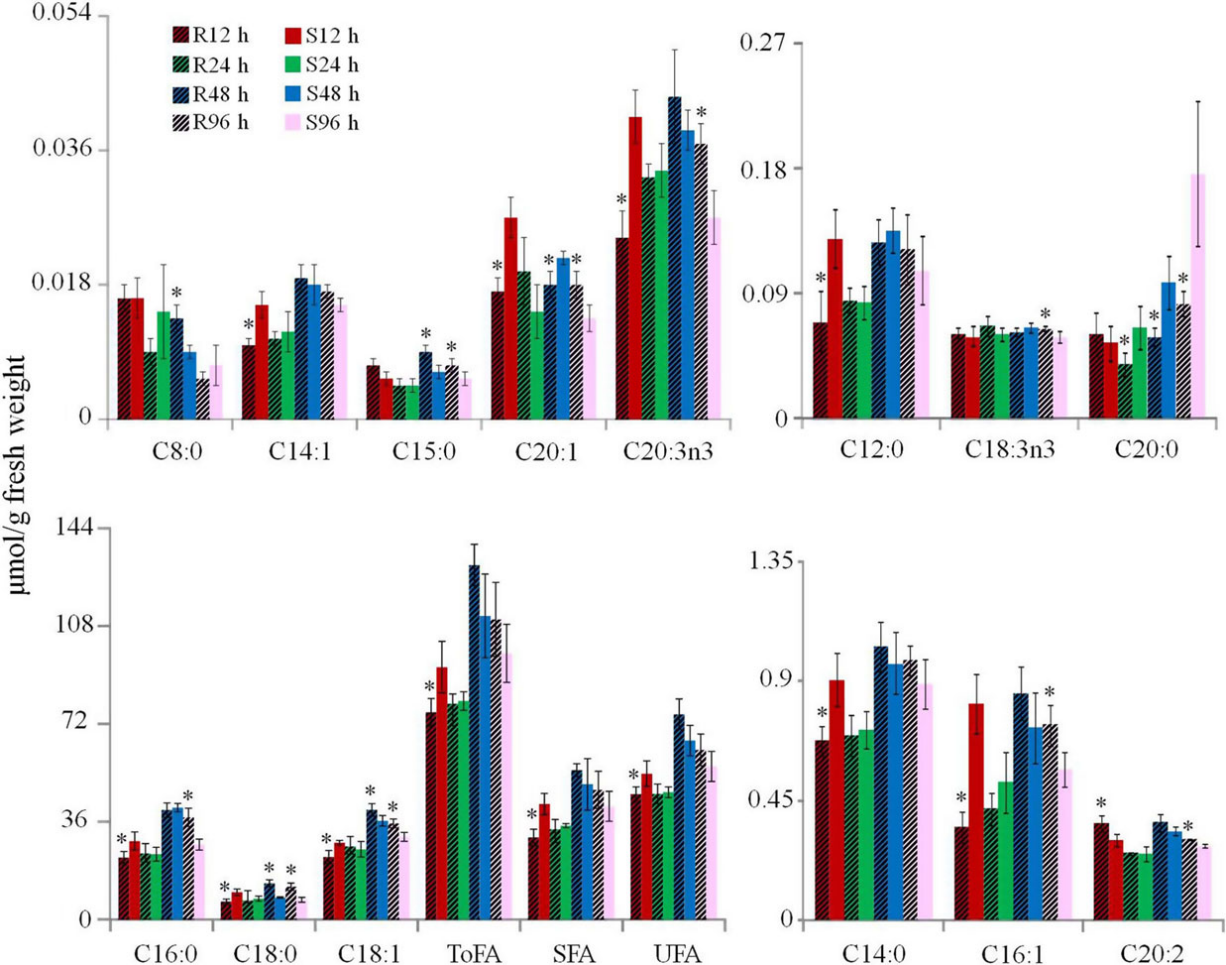
Fatty acid contents in BPH feeding on the resistant and susceptible rice plants. ToFA, total fatty acids; SFA, saturated fatty acids; UFA, unsaturated fatty acids. R12h and S12h indicate fatty acids in BPH nymphs feeding on the resistant and susceptible rice plants for 12 h, respectively. *P < 0.05 indicated statistically significant differences in R12h vs S12h, R24h vs S24h, R48h vs S48h or R96h vs S96h.

Fatty acids in BPH nymphs mainly consist of palmitic acid (C16:0), stearic acid (C18:0) and its unsaturated forms oleic acid (C18:1n9) and linoleic acid (C18:2n6), accounting for over 98% of total fatty acids (Table S2). This has not been reported previously to the best of our knowledge. Marked differences in fatty acid profiles were observed for BPH nymphs feeding on susceptible and resistant rice lines. After initial feeding for 12 h, the levels of all fatty acids were lower in BPH nymphs infesting the resistant rice plants compared with those infesting the susceptible rice plants with only exception for eicosadienoic acid (C20:2) whose level was significantly higher in BPH infesting the resistant plants (Figure 4, Table S2). However, no significant differences were observed for most fatty acids at 24 h between BPH nymphs feeding on two different regimes with exception for eicosanoic acid (C20:0) showing lower level in BPH nymphs feeding on resistant plants (Table S2). After feeding for 48 h on BPH‐resistant plants, the levels of C8:0, C15:0, C18:0 and C18:1 were higher, while the levels of C20:0 and C20:1 were lower than those feeding on the susceptible plants (Figure 4, Table S2). After feeding for 96 h on resistant plants, most BPH fatty acids had significantly higher levels including SFA (C15:0, C16:0 and C18:0) and UFA (C16:1, C18:1, C18:3n3, C20:1, C20:2 and C20:3n3) (Figure 4, Table S2).

### Quantitative real‐time PCR analysis of gene expression

**Figure 5:**
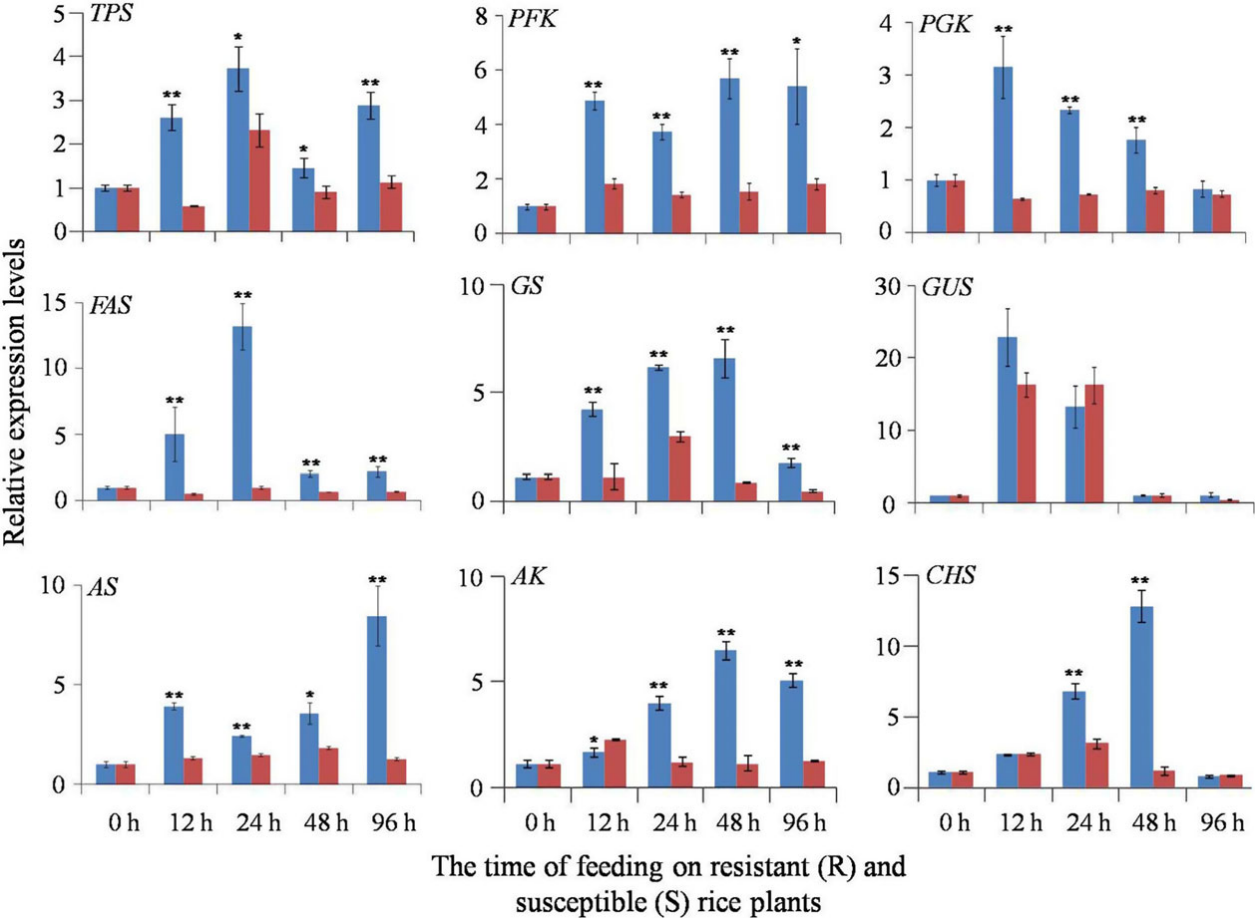
Quantitative real‐time PCR results for the mRNA expression levels of *TPS* (trehalose 6‐phosphate synthase), *PFK* (phosphofructokinase), *PGK* (phosphoglycerate kinase), *FAS* (fatty acid synthase), *GS* (glutamine synthase), *GUS* (glutamate synthase), *AS* (asparagine synthase), *AK* (arginine kinase) and *CHS* (chitin synthase) in BPH nymphs. BPH *actin1* was used as the reference gene. Expression of genes was quantified relative to the value obtained from 0 h (controls). Asterisk indicated statistically significant differences between BPH nymphs feeding on resistant rice plants (R, blue bars) and ones feeding on susceptible rice plants (S, red bars). *, *P* < 0.05; **, *P* < 0.01.

The expression levels of nine key genes were determined, including those associated with the biosyntheses of trehalose, amino acids, chitin, fatty acids as well as glycolysis (Figure 5). Trehalose, the main blood sugar, is synthesized in the fat body of insects catalysed by trehalose‐6‐phosphate synthase (TPS) (Gu *et al*., 2009). We found that the expression level of the *TPS* gene was significantly higher in BPH nymphs feeding on the resistant plants than in those feeding on the susceptible plants at all time points (Figure 5). In insects, glycolysis produces precursors for biosyntheses of amino acids, fatty acids, TCA cycle intermediates generating ATP and NADH, where phosphofructokinase (PFK) and phosphoglycerate kinase (PGK) are two key enzymes. The expression levels of *PFK* and *PGK* genes were significantly higher in BPH nymphs feeding on the resistant plants than in those feeding on the susceptible plants at all time points (Figure 5). Fatty acids are important energy storage in insects (Stanley‐Samuelson *et al*., 1988) with fatty acid synthase (FAS) as a key enzyme for fatty acid biosynthesis. Our results showed that *FAS* gene expression had trend similar to *TPS*,* PFK* and *PGK* genes (Figure 5).

Glutamine affords nitrogen sources for the biosynthesis of amino acids, pyrimidines and purines. Glutamine synthase (GS) catalyses the conversation of glutamate to glutamine (Gln) with Gln and GS playing some important roles in the fecundity of insects (Fu *et al*., 2015; Zhai *et al*., 2013, 2015). In this study, the expression of the *GS* gene followed the same trend as the *TPS* gene (Figure 5). Similar responses were observed for biosynthesis of Asn from Asp with the expression of *AS* gene encoding asparagine synthase (AS) having similar behaviour to *TPS*,* PFK*,* PGK, FAS* and *GS* genes (Figure 5). No significant differences were observed for *GUS* gene (glutamate synthase) (Figure 5).

In insect energy metabolism, phosphoarginine is the sole donor affording phosphate group for ATP production, and arginine kinase (AK) is sole kinase for arginine phosphorylation (Brown and Grossman, 2004; Tanaka *et al*., 2007). The *AK* gene was initially down‐regulated at 12 h of feeding on resistant rice plants, but subsequently up‐regulated from 24 h of feeding onwards (Figure 5). Chitin is a major constituent of insect cuticles playing significant roles in insect development and protecting insects from biotic and abiotic stresses (Moussian, 2010). In insects, chitin is synthesized by chitin synthase (CHS), which is a highly conserved enzyme found in all chitin‐synthesizing organisms (Merzendorfer, 2011). In this study, the expression level of the *CHS* gene was significantly increased after 24 h and 48 h of BPH feeding on resistant rice plants compared with those feeding on susceptible plants. After 96 h of feeding, the expression of this gene became the same for BPH feeding on both plants (Figure 5).

## Discussion

**Figure 6:**
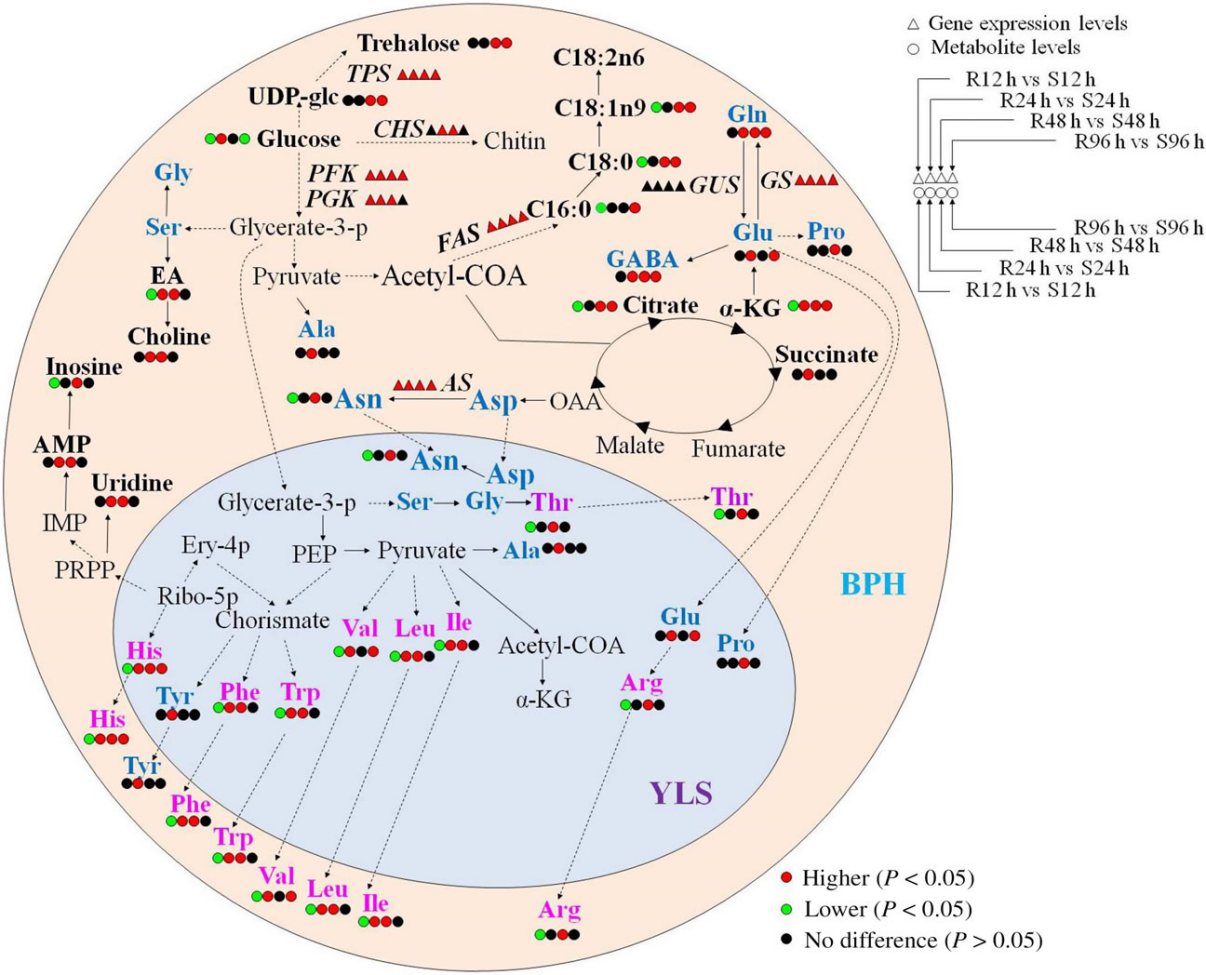
Metabolic changes in BPH nymphs feeding on resistant rice plants compared with those in BPH nymphs feeding on susceptible lines. R12h, R24h, R48h and R96h indicate metabolites in BPH nymphs feeding on resistant rice plants for 12 h, 24 h, 48 h and 96 h, respectively. S12h, S24h, S48h and S96h indicate metabolites in BPH nymphs feeding on susceptible rice plants for 12 h, 24 h, 48 h and 96 h, respectively. R12h vs S12h indicates that the changed metabolites or genes in BPH nymphs feeding on resistant rice plants for 12 h compared with those in BPH nymphs feeding on susceptible lines for 12 h. The pale yellow and pale blue areas represent BPH and endosymbiont cell, respectively. Essential amino acids are represented by rose red, and nonessential amino acids by dark blue. Metabolites identified and genes whose transcription profiles were examined were shown in bold letters. UDP‐glc, uridine diphosphate glucose; glycerate‐3‐p, glycerate‐3‐phosphate; PEP, phosphoenolpyruvate; EA, ethanolamine; Ribo‐5p, ribulose‐5‐phosphate; Ery‐4p, erythrose‐4‐phosphate; α‐KG, α‐ketoglutarate; OAA, oxaloacetate; GABA, γ‐aminobutyrate; PRPP, phosphoribosyl pyrophosphate; IMP, inosine monophosphate; AMP, adenosine monophosphate. *TPS*, trehalose 6‐phosphate synthase; *PFK*, phosphofructokinase; *PGK*, phosphoglycerate kinase; *FAS*, fatty acid synthase; *GS*, glutamine synthase; *GUS*, glutamate synthase; *AS*, asparagine synthase; YLS, yeast‐like symbionts.

BPH is a destructive pest for rice plants, and understanding the complex BPH–plant interactions is beneficial to development of environment‐friendly BPH‐combating strategies. In our previous report, we studied the metabolic responses of the resistant and susceptible plants to BPH feeding (Liu *et al*., 2010). Investigating the impacts of resistant and susceptible rice plants on BPH is also highly important for comprehending the complex interactions between rice and BPH. In this work, we employed metabonomics to investigate the differential metabolic responses of BPH towards the resistant and susceptible plants with the combined global metabonomic and targeted transcriptomic approaches based on quantitative real‐time PCR (Figure 6). BPH showed significantly different metabolic responses towards the resistant and susceptible rice plants, including glycolysis and metabolisms of amino acids, trehalose, fatty acids and chitin (Figure 6).

### BPH amino acid metabolism

The rice phloem sap is the sole nutrition source for BPH providing various amino acids, such as Gly, Glu, Gln, Asp, Asn, Ser and Ala. BPH development was severely impaired when transferred from the susceptible rice to the resistant rice for 72 h (Chen *et al*., 2011b; Sõgawa and Pathak, 1970). In this study, we found that all 19 amino acids detected in BPH nymphs had lower levels after 12 h of feeding on the resistant rice plants than those feeding on the susceptible line (Figures 2, 3 and Tables 1, 2). This deficiency of amino acids at early feeding stage was probably due to the reduced BPH uptakes of phloem sap caused by callose coated on the sieve plates of the resistant rice plants as recorded (Hao *et al*., 2008). In the meantime, the significantly higher expression of chitin synthase in BPH nymphs feeding on the resistant rice (Figures 5 and 6) demanded more amino acids for biosynthesis of this enzyme and amino groups (from Gln) for biosynthesis of the chitin precursor (N‐acetyl‐glucosamine). Such requirements are critical for the survival of BPH as chitin is an essential component of the insect cuticle for exoskeleton, peritrophic membrane that lines the mid‐gut epithelium together with the cuticular lining of foregut, hindgut and tracheae (Arakane *et al*., 2008). Such vital requirement may also explain lower level of glucose in the BPH feeding on the resistant rice line because glucose is the precursor of N‐acetyl‐glucosamine for chitin biosynthesis.

To survive from such lack of nutrients, BPHs have to quickly adapt to this adverse growing condition by hopping more frequently to ingest more sap from plants (Hao *et al*., 2008). In addition, YLS harboured in BPH may have a critical role in providing some essential amino acids for their hosts (Chen *et al*., 1981; Cheng *et al*., 2013; Koyama, 1985; Pang *et al*., 2012) because BPH's sole food source, rice phloem sap, may not provide sufficient nutrients (Hayashi and Chino, 1990). This notion was supported by the fact that the growth of aposymbiotic nymphs was negatively influenced when any of the essential amino acids (such as Thr, His, Ile, Phe or Arg) was deficient in the artificial diet (Fu *et al*., 2001). Decreased levels of essential amino acids (such as His, Ile, Leu and Phe) could be compensated by the increased number of YLS (Wang *et al*., 2004). Our observed recovery of the levels of essential amino acids (Ile, Leu, Val, Phe, Trp and His) after 24 h of feeding on the resistant rice together with significantly higher expression levels of glutamine synthase gene and asparagine synthase gene in BPH nymphs feeding on the resistant rice variety (Figure 6) was consistent with the fact that YLS might play an important role in providing some essential amino acids for their hosts. Hong *et al*. (2008) reported that the majority of the free amino acids in BPH honeydew increased when BPH was transferred from the susceptible rice TN1 to the resistant rice IR26 (Hong *et al*., 2008). Fewer amino acids are altered as feeding persisted, suggesting that BPH insects were adapting to the resistant rice lines.

### BPH trehalose metabolism

Trehalose is the main blood sugar of insects and the main energy source for hopping (Gu *et al*., 2009). Trehalose is synthesized from glucose via trehalose 6‐phosphate synthase (TPS). In the current investigation, we found significantly higher expression level for *TPS* gene (Figure 5) and higher trehalose level together with reduced levels of glucose in BPH nymphs feeding on the resistant rice variety (Figure 2, Table 2). These observations are broadly consistent with previous reports that BPHs feeding on resistant rice plants hop more frequently than those on susceptible plants (Hao *et al*., 2008). In this study, we also found significantly higher expression of arginine synthase in BPH feeding on the resistant rice variety (Figure 5) to meet the demands for more trehalose and ATP as energy sources of their hopping than in those feeding on susceptible plants.

### BPH fatty acid metabolism

Fatty acids serve as energy stores in insects (Stanley‐Samuelson *et al*., 1988), which are dominated by palmitic acid (C16:0), stearic acid (C18:0), oleic acid (C18:1n9) and linoleic acid (C18:2n6). Similar to the amino acid changes, we noted decreased levels of fatty acids in BPH at the early time point of feeding on resistant plants (Figure 4, Table S2). This observation further supports the notion that BPH growth is not favoured at early stage of feeding on resistant plants, which is consistent with previous investigations (Alagar *et al*., 2007). With prolonged feeding on the resistant rice plants, higher levels of fatty acids together with higher expression of fatty acid synthase gene than on susceptible rice line indicated that BPHs were able to adapt to a less optimum feeding condition with promoted amino acid synthesis and lipid storage. This is further supported by the fact that the changes in TCA cycle intermediates (α‐ketoglutarate and citrate) had trend similar to the amino acids and lipids together with higher expression of phosphofructokinase and phosphoglycerate kinase genes in BPH fed on the resistant rice (Figure 5, Table 2).

Peng *et al*. (2016) reported that the BPH honeydew feeding on a BPH‐resistant rice line, YHY15, for 48 h had higher levels for two TCA cycle intermediates (succinate and malate) and urea but lower levels for six amino acids (Val, Leu, Ser, Thr, Pro and Gln) than those feeding on a susceptible line, TN1. In this study, we also found that BPHs feeding on the resistant line for 48 h differed significantly in their metabolic phenotypes from those feeding on susceptible rice plants. The former had higher levels for 11 amino acids (Thr, Pro, Gln, Ile, Leu, Arg, GABA, Asn, Phe, Tyr and His), four fatty acids (C8:0, C15:0, C18:0 and C18:1n9), two TCA cycle intermediates (α‐ketoglutarate and citrate), five nucleosides/nucleotides (AMP, UDP‐glucose, inosine, NAD and uridine), three choline metabolites (dimethylamine, ethanolamine and choline), formate and trehalose but lower levels for dAMP, C20:0 and C20:1 than the latter (Figure 6). This is not surprising because honeydew can partially reflect the BPH utilization of rice phloem sap (Sõgawa and Pathak, 1970), whereas metabolites in BPH reflect the insect's whole body metabolism apart from dietary nutrients.

Our previous study showed that BPH feeding induced stronger up‐regulations of both GABA shunt and shikimate‐mediated secondary metabolism in the BPH‐resistant rice plants than in the susceptible ones (Liu *et al*., 2010). The activation of GABA shunt served as a rapid and effective way to alleviate the intracellular hyperammonia resulting from BPH invasion caused by reactive oxygen species (ROS) generation; activation of the shikimate‐mediated metabolism probably promoted biosynthesis of plant secondary metabolites acting as antioxidants and BPH deterrents (Liu *et al*., 2010). The latter is expected to discourage BPHs from ingesting rice sap and BPHs will have to adopt a strategy to obtain sufficient essential nutrients. This study showed that initial feeding on the resistant plants caused nutrient deficiency for BPHs (e.g. amino acids, glucose and fatty acids) compared with feeding on the susceptible ones. BPHs showed partial recovery from such deficiency after 24 h of feeding (Figure 6) probably by ingesting more sap from rice plants and obtaining nutrients from YLS. These results indicated that both BPHs and rice plants had their own ways of adaptive actions towards each other with fairly different strategies. Therefore, it can be proposed that ultimate development of effective BPH‐resistant rice requires concurrent enhancement of the shikimate‐mediated secondary metabolisms in rice plants and impairment of YLS in BPH.

To sum up, there were significant metabolic differences between BPH nymphs feeding on the resistant rice and susceptible rice lines and such differences were dynamically dependent on the feeding processes. In the early stage of feeding on resistant rice plants, BPH nymphs suffered from nutrient deprivation causing deficiency of amino acids, fatty acids, glucose and subsequent energy metabolism. To survive from this plant resistance, BPH nymphs probably ingest more sap from rice with increased hopping frequency and/or get necessary nutrients from their yeast‐like symbionts at later feeding stages. This indicates that rice resistance to BPH is complex and associated with both BPH–rice and BPH–YLS interactions. BPH–YLS interactions are potentially important for BPH to overcome rice plant resistance. It, nevertheless, remains to be fully understood whether the observations obtained from the biotype 1 BPH and resistant rice line carrying *Bph15* here are generic for other BPH biotypes and/or resistant rice varieties carrying other BPH‐resistant genes.

## Experimental procedures

### Chemicals

Methanol, K_2_HPO_4_ and NaH_2_PO_4_ were purchased from Guoyao Chemical Co. Ltd. (Shanghai, China), whereas D_2_O (99.9% D) and sodium 3‐trimethylsilyl [2, 2, 3, 3‐D4] propionate (TSP) were from Cambridge Isotope Laboratory (Miami, FL). A mixture of 37 standards for methyl esters of fatty acids and hexane were from Supelco (Bellefonte, PA).

### Plant materials and insects

Two rice lines were used in this study: a BPH‐susceptible line and a BPH‐resistant line. The former, Taichung Native 1 (TN1), is a conventional variety with no BPH resistance genes, while the latter is a near‐isogenic line carrying *Bph15* gene (NIL‐*Bph15*) in TN1 genetic background, which exhibits high BPH resistance derived from YHY15/TN1F1 plants back‐crossed four times with TN1 through marker‐assisted selection (Lv *et al*., 2014). All rice plants were grown in pots with each containing 10 plants in the glasshouse (70%–80% R.H., 25 °C, 16‐h light/8‐h dark) at the Institute of Genetics, Wuhan University (Wuhan, China). The biotype 1 BPH insects were originally maintained on TN1 rice plants in the glasshouse. A total of 44 pots of each rice variety were maintained in glasshouse to represent 11 biological replicates (*n* = 11). After three weeks, a total of 80 s‐ and third‐instar BPH nymphs were introduced to each pot. BPH samples were then collected at 12 h, 24 h, 48 h and 96 h after feeding with the survived nymphs from each pot collected as one sample. These samples were immediately frozen in liquid nitrogen and kept at −80 °C for further metabolite and mRNA analyses.

### BPH metabolite extraction

Each sample of BPH nymphs was individually ground well in liquid nitrogen into fine powder. About 80 mg of such powder was transferred into an Eppendorf tube followed by an addition of 600 μL CH_3_OH/H_2_O (v/v = 2:1) and a 5‐mm tungsten carbide bead (Qiagen, Germany). After vortexing, the mixture was homogenized using a TissueLyser (Qiagen, Germany) followed by 15‐min intermittent sonication in an ice bath. The supernatant was collected after centrifugation for 10 min (16 099 ***g***, 4 °C). This extraction procedure was further repeated twice and these supernatants from three extractions were combined as one sample. The insoluble residues were collected for fatty acid analysis. After removal of methanol under vacuum, samples were lyophilized. The freeze‐dried extracts were redissolved in 600 μL phosphate buffer (0.1 M K_2_HPO_4_–NaH_2_PO_4_, pH 7.4) containing 90% D_2_O and 0.002% TSP (Xiao *et al*., 2009). Following 10 min of centrifugation (16 099 ***g***, 4 °C), 550 μL of supernatant from each sample was collected into a 5‐mm NMR tube for metabolite analysis.

### NMR spectroscopy

All ^1^H NMR spectra were acquired on a Bruker AVIII 600 spectrometer (600.13 MHz for ^1^H) at 298 K equipped with an inverse detection cryogenic probe (Bruker BioSpin, Germany). A standard first increment of NOESY pulse sequence (RD‐90°‐t1‐90°‐tm‐90°‐acquisition) was used to acquire ^1^H NMR spectra. 90° pulse length was 10 μs and t1 was 3 μs. Water peak was saturated with a continuous wave irradiation during the recycle delay (RD) of 2 s and mixing time (tm) of 80 ms. 64 transients were collected into 32 k data points with a spectral width of 20 ppm. A set of 2D NMR spectra including ^1^H‐^1^H TOCSY, ^1^H‐^1^H COSY, ^1^H‐JRES, ^1^H‐^13^C HSQC and ^1^H‐^13^C HMBC spectra were acquired for selected samples to unambiguously assign resonances (Li *et al*., 2015; Liu *et al*., 2016).

### Spectral processing and data analysis

After phase and baseline collection, all spectra were referenced to TSP at δ 0.00. NMR spectral region between 0.5 and 10.0 ppm was divided into segments of 0.004 ppm (2.4 Hz) using AMIX (v3.9.2, Bruker BioSpin) with water region at δ 4.678–5.170 discarded. The areas of all remaining buckets were normalized to the fresh weight of BPH nymph powder. PCA and OPLS‐DA (Trygg and Wold, 2002) were carried out on the normalized NMR data using SIMCA‐P+ software (v12.0, Umetrics, Sweden). The qualities of OPLS‐DA models were assessed with CV‐ANOVA approach taking *P* < 0.05 as significant (Eriksson *et al*., 2008). The results were presented as loadings plots, after back‐transformation, with colour‐coded absolute values of the correlation coefficients (|*r*|) (Cloarec *et al*., 2005). In such plots, variables (i.e. metabolites) with warm colour (e.g. red) show more significant contributions to intergroup differences than those with cool colour (e.g. blue). In our study, the metabolites exhibiting statistically significant changes were acquired at the level of *P* < 0.05. The altered metabolites were also presented by relative concentration changes as calculated in the forms of (*C*_*R*_–*C*_*S*_)/*C*_*S*_, where *C*_*R*_ and *C*_*S*_ stood for the concentration of metabolites in BPH nymphs fed on resistant and susceptible rice plants, respectively.

### Quantification of BPH metabolites

The metabolites in BPH fed on the resistant and susceptible rice plants were quantified by calculating from integrals of the (clean and less overlapped) NMR resonances from the concerned metabolites and internal reference, TSP, with known concentration. This was performed by taking their relaxation time (T_1_) into consideration as described previously (Chen *et al*., 2011a).

### GC‐FID/MS analysis of fatty acids in BPH insects

BPH fatty acids were quantitatively measured using a previously reported method (Liu *et al*., 2016) with some minor modifications. In brief, 500 μL HPLC‐grade CH_3_OH was added to 20 mg of BPH residues from polar metabolite extraction. Methylation and GC‐MS detection were conducted as previously reported (Liu *et al*., 2016). Fatty acid identification was achieved by comparing to a mixture of standard compounds and further confirmed by comparing their mass spectrometry data with databases. The results were expressed as micromole fatty acids per gram fresh weight of BPH nymph powder.

### RNA extraction and cDNA synthesis

Total RNA was extracted from about 20 mg samples of BPH nymphs with TRIzol reagent (Invitrogen). First‐strand cDNA was synthesized using 1 μg of total RNA with a Primer Script RT reagent Kit (TaKaRa, Dalian, China) following the manufacturer's instructions.

### Quantitative real‐time PCR analysis

The primers used in real‐time PCR are listed in Table S3. A total of 2 μL of the synthesized first‐strand cDNA was amplified by PCR in 20 μL reaction mixtures using Sybr Select Master Mix (Applied Biosystems) on a Step One Real‐time PCR system (Applied Biosystems) with the following procedure: 50 °C for 2 min and 95 °C for 2 min, followed by 35–45 cycles of 95 °C for 15 s, 60 °C for 1 min; *actin 1* gene was used as the reference gene. Melting curve analyses were performed to ensure that the PCR products were unique. The values were averaged using three independent biological samples, and relative expression levels of selected genes were analysed by 2 Ct method (Livak and Schmittgen, 2001).

## Supporting information


**Figure S1** PCA scores plots between BPH nymphs feeding on the resistant rice plants (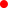) and ones feeding on the susceptible lines (■) for (a) 12 h, (b) 24 h, (c) 48 h and (d) 96 h.
**Table S1** Assignments of NMR data for metabolites in BPH nymphs feeding on resistant and susceptible rice plants.
**Table S2** Composition of fatty acids of BPHs feeding on the resistant and susceptible rice plants (μmol/g).
**Table S3** Primers for quantitative real‐time PCR analysis of selected genes.
