# Supplementary material for: Dynamic metabolic responses of brown planthoppers towards susceptible and resistant rice plants
Source: Plant Biotechnol J. 2017 Apr 13;15(10):1346–57. doi: 10.1111/pbi.12721 (PMC5595709; doi:10.1111/pbi.12721)
Supplement: Supplementary file 1 — Figure S1 PCA scores plots between BPH nymphs feeding on the resistant rice plants () and ones feeding on the susceptible lines (■) for (a) 12 h, (b) 24 h, (c) 48 h and (d) 96 h. Table S1 Assignments of NMR data for metabolites in BPH nymphs feeding on resistant and susceptible rice plants. Table S2 Composition of fatty acids of BPHs feeding on the resistant and susceptible rice plants (μmol/g). Table S3 Primers for quantitative real‐time PCR analysis of selected genes. [file PBI-15-1346-s001.pdf]

Supplementary information

Dynamic metabolic responses of brown planthoppers towards susceptible and resistant rice plants

Caixiang Liu[1,†], Ba Du[3,†], Fuhua Hao[1], Hehua Lei[1], Qianfen Wan[1,2], Guangcun He[3], Yulan Wang[1,4] and Huiru Tang[1,2*]*

[1]CAS Key Laboratory of Magnetic Resonance in Biological Systems, State Key Laboratory of Magnetic Resonance and Atomic and Molecular Physics, National Centre for Magnetic Resonance in Wuhan, Wuhan Institute of Physics and Mathematics, the Chinese Academy of Sciences, Wuhan 430071, China

[2]State Key Laboratory of Genetic Engineering, Zhongshan Hospital and School of Life Sciences, Fudan University, Collaborative Innovation Center for Genetics and Development, Metabonomics and Systems Biology Laboratory at Shanghai International Centre for Molecular Phenomics, Shanghai, 200438, China

[3]State Key Laboratory of Hybrid Rice, College of Life Sciences, Wuhan University, Wuhan 430072, China

[4]Collaborative Innovation Center for Diagnosis and Treatment of Infectious Diseases, Zhejiang University, Hangzhou 310058, China

[*]To whom all correspondence should be addressed:

Huiru Tang: Tel, +86-21-51630725; fax, +86-21-51630381; Email, huiru_tang@fudan.edu.cn

Yulan Wang: Tel, +86-27-87197143; fax, +86-27-87199291; Email, yulan.wang@wipm.ac.cn

[†]These authors contributed equally to this work.

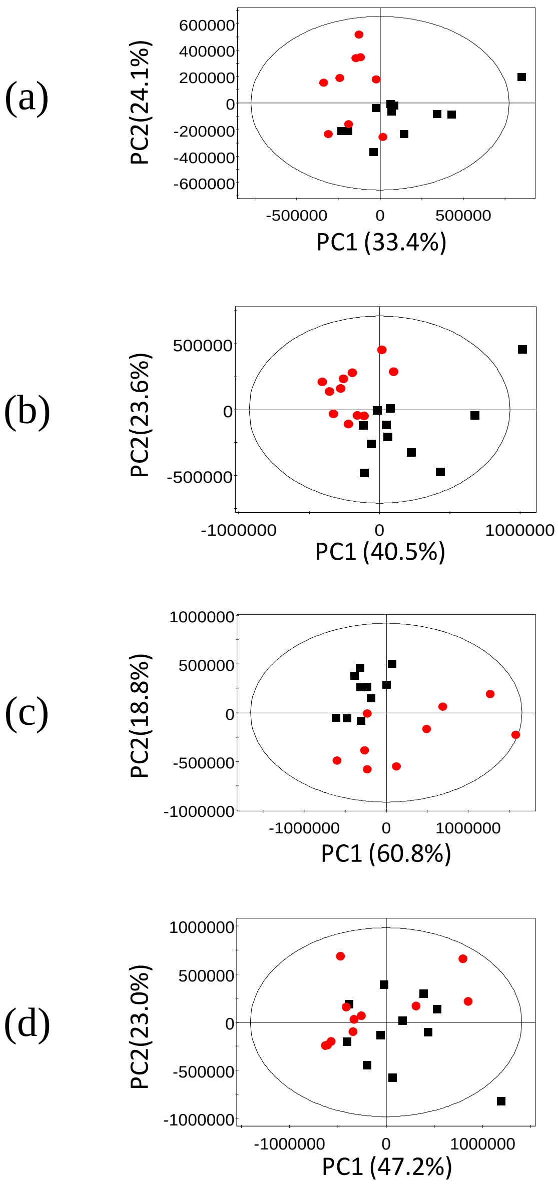

Figure S1. PCA scores plots between BPH nymphs feeding on the resistant rice plants (●) and ones feeding on the susceptible lines (■) for (a) 12 hours, (b) 24 hours, (c) 48 hours and (d) 96 hours.

Table S1. Assignments of NMR data for metabolites in BPH nymphs feeding on the resistant and susceptible rice plants

no.	metabolites	assignments	δ^{1}H (ppm)	δ^{13}C (ppm)
1	Isoleucine (Ile)	δ-CH_3	0.94 (t) [a]	13.9
		γ'-CH_3	1.01 (d)	17.4
		β-CH	1.99 (m)	38.9
2	Leucine (Leu)	δ'-CH_3	0.96 (d)	ND [b]
		δ-CH_3	0.97 (d)	26.6
		β-CH_2	1.72 (m)	42.8
3	Valine (Val)	γ'-CH_3	1.04 (d)	20.7
		γ-CH_3	0.99 (d)	19.5
		β-CH	2.27 (m)	31.7
		α-CH	3.61 (d)	63.2
4	2-ketoisovalerate	δ-CH_3	1.13 (d)	19.3
		γ-CH	3.04 (dq)	38.8
5	3-methylaspartate	δ-CH_3	1.11 (d)	19.1
		γ-CH	2.95 (m)	42.7
		β-CH	4.00 (d)	66.1
6	Threonine (Thr)	γ-CH_3	1.33 (d)	20.0
		β-CH	4.24 (m)	68.4
		α-CH	3.60 (d)	62.7
7	2-Hydroxyisobutyrate	β-CH_3	1.37 (s)	28.9
8	Lysine (Lys)	γ-CH_2	1.45 (m)	24.0
		δ-CH_2	1.73 (m)	29.5
		β-CH_2	1.92 (m)	33.2
9	Alanine (Ala)	β-CH_3	1.48 (d)	19.2
		α-CH	3.78 (q)	53.8
		COOH		178.8
10	Arginine (Arg)	δ-CH_2	3.24 (t)	42.5
		γ-CH_2	1.66 (m)	24.5
		β-CH_2	1.92 (m)	29.5
		α-CH	3.78 (t)	57.5
11	Acetate	β-CH_3	1.92 (s)	26.3
12	Proline (Pro)	δ-CH	3.37 (t), 3.41 (t)	50.0
		γ-CH_2	2.02 (m), 2.08 (m)	26.7
		β-CH	2.36 (m)	32.5
		α-CH	4.14 (t)	65.0
13	γ-aminobutyrate (GABA)	γ- CH_2	3.02 (t)	41.3
		β- CH_2	1.90 (quintet)	26.6

		α- CH_2	2.30 (t)	37.0
14	α-Ketoglutarate (α-KG)	γ- CH_2	3.02 (t)	38.9
		β- CH_2	2.44 (t)	33.5
15	Pyruvate	β-CH_3	2.38 (s)	32.0
16	Succinate	α, β-CH_2	2.41 (s)	37.3
		COOH		184.6
17	Glutamine (Gln)	γ-CH_2	2.45 (m)	33.9
		β-CH_2	2.14 (m)	29.4
		α-CH	3.78 (dd)	57.2
18	Glutamate (Glu)	γ-CH_2	2.35(m)	36.2
		β-CH	2.13(m)	29.1
		β’-CH	2.06 (m)	29.1
		α-CH	3.76 (dd)	57.6
19	Dimethylamine	N-CH_3	2.76 (s)	47.4
20	Asparate (Asp)	β-CH	2.68 (dd)	37.0
		β’-CH	2.82 (dd)	37.0
21	Asparagine (Asn)	β’-CH	2.96 (dd)	37.2
		β-CH	2.86 (dd)	37.2
		α-CH	4.01 (dd)	54.3
		COOH		176.5
22	Ethanolamine (EA)	N-CH_2	3.15 (t)	43.8
		O-CH_2	3.83 (t)	60.7
23	Choline	N-(CH_3)+	3.20 (s)	56.6
		β-CH_2	3.52 (m)	70.0
		α-CH_2	4.07 (m)	57.9
24	Glycerophosphocholine	N-(CH_3)+	3.23 (s)	56.9
	(GPC)	β-CH_2	3.68 (m)	62.1
		α-CH_2	4.33 (m)	62.2
25	Glycine (Gly)	α-CH	3.57 (s)	44.0
26	β-Glucose	C1H	4.65 (d)	98.8
		C2H	3.25 (t)	77.1
		C3H	3.49 (t)	78.7
		C4H	3.41 (dd)	72.5
		C6H	3.90	63.7
		C6’H	3.75	63.8
27	α-Glucose	C1H	5.24 (d)	94.8
		C2H	3.53	74.2
		C3H	3.70	75.6
		C6H	3.83	63.4
28	Trehalose	C1H	5.20 (d)	96.0
		C2H	3.66 (q)	78.5
		C3H	3.86 (dd)	76.0
29	Fumarate	α, β-C=C	6.52 (s)	138.1
		COOH		177.4

30	Tyrosine (Tyr)	C2, 6H, ring	7.19 (d)	133.8
		C3, 5H, ring	6.90 (d)	118.5
31	Phenylalanine (Phe)	C2, 6, ring	7.33 (m)	131.8
		C3, 5, ring	7.43 (d)	131.8
		C4, ring	7.38 (m)	130.8
		COOH		178.4
32	Tryptophan (Trp)	C7H, ring	7.53 (d)	114.8
		C6H, ring	7.28 (t)	124.8
		C5H, ring	7.20 (t)	122.1
		C4H, ring	7.73 (d)	121.3
		C2H, ring	7.33 (s)	127.1
33	Formate	HCOO-	8.46 (s)	ND
34	Adenosine monophosphate	C2H	8.61 (s)	ND
	(AMP)	C8H	8.27 (s)	ND
		C1H of ribose	6.14 (d)	89.9
		C2H of ribose	4.51 (m)	73.5
		C3H of ribose	4.37 (m)	87.6
35	Adenosine diphosphate (ADP)	C2H	8.54 (s)	ND
	/Adenosine triphosphate (ATP)	C8H	8.27 (s)	ND
36	Uridine monophosphate	C1H of ribose	5.99 (d)	90.9
	(UMP)	C3H of ribose	4.43 (t)	73.0
		C2H of ribose	4.34 (t)	72.3
37	Cytidine monophosphate	C-CH	8.10 (d)	ND
	(CMP)	N-CH	6.14 (d)	ND
		C2H of ribose	6.01 (d)	92.3
38	Uridine diphosphate	N-CH	7.96 (d)	ND
	glucose (UDP-Glucose)	C-CH	5.97 (d)	ND
		C1H of glucose	5.62 (m)	80.4
39	Guanosine	C8H	8.01 (s)	145.3
40	Inosine	C2H	8.35 (s)	143.0
		C8H	8.24 (s)	149.5
		C1H of ribose	6.10 (d)	90.8
		C3H of ribose	4.44 (dd)	73.6
		C4H of ribose	4.29 (m)	73.6
41	Nicotinamide adenine	C2H	9.34 (s)	ND
	dinucleotide (NAD)	C6H	9.15 (d)	ND
		C4H	8.83(d)	ND
		C2H of adenine	8.43 (s)	ND
		C8H	8.17 (s)	ND
42	Uridine	N-CH of uracil	7.88 (d)	144.5
		C-CH of uracil	5.92 (d)	92.3
		C2H of ribose	5.90 (d)	105.1
43	Allanotoin	CH	5.39 (s)	ND
44	Histidine (His)	C4H, ring	7.10 (s)	119.6

		C2H, ring	7.85 (s)	138.6
45	Uracil	C5H	5.80 (d)	103.8
		C6H	7.55 (d)	146.1
46	Myo-inositol	C1,3H	3.54 (dd)	74.1
		C2H	4.07 (t)	74.0
		C4,6 H	3.63 (t)	74.5
		C5H	3.28 (t)	76.4
47	Carnosine	C=CH-N of Histidine	7.09 (s)	118.5
		N=CH-N of Histidine	8.08 (s)	137.8
48	Uridine diphosphate	CH_3	2.08 (s)	24.9
	-N-acetylglucosamine	CH_2	5.52 (dd)	97.3
49	Dimethylglycine	CH_3	2.93 (s)	ND
50	U1	CH_3	2.97 (s)	ND
51	Citrate	α,γ-CH	2.55 (dd)	48.1
		α',γ'-CH	2.67 (dd)	48.1
52	U2		6.84 (d)	ND
53	Deoxyadenosine monophosphate (dAMP)	2-CH	8.52 (s)	ND

[a]Multiplicity: s, singlet; d, doublet; t, triplet; q, quartet; dd, doublet of doublets; qu, quintet; m, multiplet; c, complex; br, broad signals. [b]The signals were not determined.

Table S2. Composition of fatty acids of BPHs feeding on the resistant and susceptible rice plants (μmol/g)[a]

	R12h[b]	S12h [b]	R24h	S24h	R48h	S48h	R96h	S96h
C8:0	0.016±<br>0.002	0.016±<br>0.003	0.009±<br>0.002	0.015±<br>0.006	0.014±<br>0.002	0.009±<br>0.001	0.005±<br>0.001	0.007±<br>0.003
C11:0	0.080±<br>0.012	0.091±<br>0.013	0.091±<br>0.008	0.075±<br>0.013	0.108±<br>0.010	0.101±<br>0.007	0.115±<br>0.016	0.094±<br>0.011
C12:0	0.071±<br>0.022	0.131±<br>0.021	0.086±<br>0.009	0.085±<br>0.012	0.129±<br>0.016	0.137±<br>0.016	0.124±<br>0.025	0.108±<br>0.025
C14:0	0.683±<br>0.057	0.914±<br>0.102	0.704±<br>0.078	0.723±<br>0.072	1.040±<br>0.095	0.977±<br>0.118	0.989±<br>0.056	0.898±<br>0.095
C14:1	0.010±<br>0.001	0.015±<br>0.002	0.011±<br>0.001	0.012±<br>0.003	0.019±<br>0.002	0.018±<br>0.003	0.017±<br>0.001	0.015±<br>0.001
C15:0	0.007±<br>0.001	0.005±<br>0.001	0.005±<br>0.001	0.005±<br>0.001	0.009±<br>0.001	0.006±<br>0.001	0.007±<br>0.001	0.005±<br>0.001
C16:0	22.988±<br>2.226	29.125±<br>3.389	24.48±<br>3.192	24.227±<br>2.584	40.622±<br>2.654	41.539±<br>1.528	37.947±<br>3.398	27.794±<br>2.030
C16:1	0.353±<br>0.060	0.823±<br>0.114	0.421±<br>0.063	0.525±<br>0.115	0.863±<br>0.101	0.732±<br>0.135	0.743±<br>0.078	0.573±<br>0.068
C18:0	6.578±<br>0.983	10.100±<br>1.220	6.990±<br>0.828	7.683±<br>0.975	13.421±<br>1.318	8.105±<br>0.238	12.12±<br>1.338	7.246±<br>0.880
C18:<br>1n9	23.216±<br>2.427	28.483±<br>0.865	27.147±<br>2.811	26.038±<br>2.902	40.694±<br>2.305	36.664±<br>1.954	35.659±<br>1.657	30.678±<br>1.652
C18:<br>2n6	22.555±<br>1.462	23.269±<br>2.035	18.793±<br>1.665	20.262±<br>1.553	32.189±<br>2.566	30.89±1.<br>058	25.695±<br>2.877	24.299±<br>1.63
C18:<br>3n3	0.063±<br>0.004	0.060±<br>0.007	0.068±<br>0.007	0.062±<br>0.005	0.064±<br>0.003	0.066±<br>0.004	0.065±<br>0.002	0.060±<br>0.005
C20:0	0.062±<br>0.015	0.055±<br>0.013	0.040±<br>0.008	0.066±<br>0.015	0.060±<br>0.006	0.099±<br>0.019	0.084±<br>0.009	0.178±<br>0.053
C20:1	0.017±<br>0.002	0.027±<br>0.003	0.020±<br>0.005	0.015±<br>0.004	0.018±<br>0.002	0.022±<br>0.001	0.018±<br>0.002	0.014±<br>0.002
C20:2	0.367±<br>0.031	0.304±<br>0.025	0.252±<br>0.004	0.251±<br>0.026	0.370±<br>0.034	0.338±<br>0.017	0.306±<br>0.001	0.281±<br>0.006
C20:<br>3n3	0.025±<br>0.004	0.041±<br>0.004	0.033±<br>0.002	0.034±<br>0.004	0.044±<br>0.006	0.039±<br>0.003	0.037±<br>0.003	0.027±<br>0.004
ToFA	77.021±<br>5.125	93.814±<br>9.589	80.181±<br>8.252	81.203±<br>3.436	131.799<br>±7.716	112.842<br>±15.591	111.544<br>±13.733	98.925±<br>10.825
SFA	30.455±<br>3.065	42.891±<br>3.949	33.51±<br>4.981	34.833±<br>0.730	55.518±<br>2.346	50.244±<br>9.576	48.184±<br>6.906	42.036±<br>5.557
UFA	46.565±<br>2.708	54.153±<br>4.744	46.672±<br>3.660	47.292±<br>1.983	76.281±<br>5.725	66.493±<br>5.642	63.037±<br>5.913	56.89±<br>5.599
SFA<br>/ToFA	0.359±<br>0.017	0.392±<br>0.006	0.379±<br>0.021	0.380±<br>0.006	0.384±<br>0.011	0.392±<br>0.021	0.397±<br>0.008	0.385±<br>0.014

UFA /ToFA	0.550± 0.017	0.525± 0.017	0.530± 0.021	0.529± 0.005	0.525± 0.011	0.525± 0.032	0.515± 0.016	0.524± 0.014

Data expressed as mean ± SD. [a]μmol per gram BPH fresh weight. [b]R12h and S12h: BPH nymphs feeding on the resistant and susceptible rice plants for 12 hours respectively. Data in red and blue respectively denote significant increase and decrease in BPH feeding on the resistant rice plants compared with these feeding on the susceptible rice plants ($p < 0.05$); ToFA, total fatty acids; SFA, saturated fatty acids; UFA, unsaturated fatty acids.

Table S3. Primers for quantitative real-time PCR analysis of selected genes

Gene Bank Accession	Gene Name	Primer sequences (5'-3')	Product length
KC445137	*Glutamine synthase*	F:TATGCCAGGGATGTGGTTGAG<br>R:CGGTGGAGAAGTTGCAGTGAG	265 bp
JQ743627	*Trehalose synthase*	F:TTGCCAAAGACTGAGGCGAATG<br>R:CCTCATCAGCCCAAGGGAACAA	196 bp
JQ040014	*Chitin synthase*	F:CCGCAAACGATTCCTACAGA<br>R:AGGTCCTTGACGCTCATTCC	222 bp
KU365925	*Arginine kinase*	F:ACCACAACGACAACAAGACCTTCC<br>R:TGGGACAGAAAGTCAGGAATCCCA	186 bp
Li et al., 2016	*Fatty acid Synthase*	F:CGGAGACTCTGCCCTAA<br>R:CAGCGACTAATCCAACATC	193 bp
Zhai et al., 2013	6-Phosphofructokinase	F:AGGCATCGCCGTCTTCACC<br>R:AGAGCCGTCTCCGCCAATC	-
Zhai et al., 2013	Phospholycerate Kinase	F:CCCACCACCCCTCAAACT<br>R:GTCAATGGCGGCCACTAG	-
JX125594	Glutamate Synthase	F:TTTCGGCCAAAGACAAGCAC<br>R:GGGTCCTTACCGGTGTTCTG	223 bp
JX125588	Asparagine Synthase	F:TAGGATGCGCTGTGTGGTTT<br>R:GTGTAGCCCCCAAGCAGTTC	127 bp
KU196668	*Actin 1*	F:TGGACTTCGAGCAGGAAATGG<br>R:ACGTCGCACTTCATGATCGAG	199 bp
